# Public Place Smoke-Free Regulations, Secondhand Smoke Exposure and Related Beliefs, Awareness, Attitudes, and Practices among Chinese Urban Residents

**DOI:** 10.3390/ijerph10062370

**Published:** 2013-06-07

**Authors:** Tingzhong Yang, Abu S. Abdullah, Li Li, Ian R. H. Rockett, Yan Lin, Jun Ying, Wei Guo, Dan Wu, Mu Li

**Affiliations:** 1 Center for Tobacco Control Research, Zhejiang University School of Medicine, Hangzhou 310058, China; E-Mails: li19891031@126.com (L.L.); beargw@gmail.com (W.G.); wudan.tracy@yahoo.com (D.W.); 2 School of Public Health, Guangxi Medical University, Nanning 530021, China; E-Mail: abu.s.abdullah@gmail.com; 3 Boston Medical Center, Boston University Medical Campus, Boston, MA 02118, USA; 4 Injury Control Research Center and Department of Epidemiology (School of Public Health), West Virginia University, Morgantown, WV 26506, USA; E-Mail: irockett@hsc.wvu.edu; 5 International Union Against Tuberculosis and Lung Disease, Beijing 100005, China; E-Mail: ylin@theunion.org; 6 Department of Environmental Health, University of Cincinnati, Cincinnati, OH 45221, USA; E-Mail: yingj@ucmail.uc.edu; 7 Sydney School of Public Health, University of Sydney, NSW 2006, Australia; E-Mail: mu.li@sydney.edu.au

**Keywords:** tobacco control, smoke-free regulations, secondhand smoke

## Abstract

*Objective:* To evaluate the association between smoke-free regulations in public places and secondhand smoke exposure and related beliefs, awareness, attitudes, and behavior among urban residents in China. *Methods:* We selected one city (Hangzhou) as the intervention city and another (Jiaxing) as the comparison. A structured self-administered questionnaire was used for data collection, and implemented at two time points across a 20-month interval. Both unadjusted and adjusted logistic methods were considered in analyses. Multiple regression procedures were performed in examining variation between final and baseline measures. *Results:* Smoke-free regulations in the intervention city were associated with a significant decline in personal secondhand smoke exposure in government buildings, buses or taxis, and restaurants, but there was no change in such exposure in healthcare facilities and schools. In terms of personal smoking beliefs, awareness, attitudes, and practices, the only significant change was in giving quitting advice to proximal family members. *Conclusions:* There was a statistically significant association between implementation of smoke-free regulations in a city and inhibition of secondhand tobacco smoking exposure in public places. However, any such impact was limited. Effective tobacco control in China will require comprehensive laws implemented fully and supported by penalties and a combination of strong public health education.

## 1. Introduction

Globally, the tobacco-smoking pandemic accounts for approximately 5.4 million deaths annually, including the deaths of more than 600,000 nonsmokers [1]. The International Labour Organization (ILO) estimates that at least 200,000 workers die every year from exposure to secondhand tobacco smoke [2]. China leads the world in tobacco consumption, and approximately one million Chinese die annually from tobacco-related diseases [3]. High smoking prevalence means that exposure to secondhand tobacco smoke in public places is common, and it characterizes most Chinese restaurants, schools, hospitals, government buildings, and train stations [4,5]. Smoke-free policies are the most effective way to reduce exposure to tobacco smoke among the public [6]. Furthermore, smoke-free laws may substantially reduce smoking prevalence, as supported not only empirically [6,7,8], but also by social norm [6,9] and behavioral susceptibility theory [10,11].

Exposure to secondhand tobacco smoke causes many serious diseases. However, millions of nonsmokers remain exposed to secondhand smoke in their homes, workplaces, public places, and vehicles. In order to combat the global spread of tobacco use and secondhand tobacco smoke exposure, the World Health Organization (WHO) established the Framework Convention on Tobacco Control (FCTC) in 1999. This framework was fully endorsed by member states on 21 May 2003. The Chinese National People’s Congress ratified the FCTC on 27 August 2005. The FCTC provides clear guidance through Article 8 (Protection from Exposure to Tobacco Smoke), which outlines specific measures and approaches for reducing population-wide tobacco smoke exposure [12].

Many studies have shown that public-place smoking restrictions are a most effective way to reduce exposure to passive tobacco smoking [6,7]. Numerous countries have approved legislation for smoke-free public places. More than 739 million people worldwide are now protected by comprehensive, national smoke-free laws, an increase of more than 385 million since 2008 [1].In ratifying the FCTC, the Chinese government agreed that all workplaces and public places should be smoke-free by 2011. To meet this objective, efforts were made to expand the number of smoke-free places throughout the country. Although China lacks a comprehensive smoke-free law, several national laws and policies regulate smoking in public places. On 1 May 2011, the Chinese Ministry of Health released “*Guidelines on the Regulatory Measures of the Sanitary Administration in Public Places*”, an action which indeed strengthened control measures on secondhand smoke in public places [13,14,15]. In recent years, the Ministry of Health and the Ministry of Education developed guidelines for making schools and hospitals smoke-free. While the tobacco control measures are intended to cover the whole nation, their effects appear limited. Unmet public expectations have motivated many local governments to initiate their own smoke-free policies or regulations. Consequently, nearly half of the Chinese midsize and large cities had instituted them by the end of 2010 [13]. Although smoke-free laws may substantially reduce secondhand smoke exposure and smoking prevalence [6,7,8,9,10,11], there is a lack of empirical evidence as to such effectiveness in China.

Public-place smoking restrictions are dedicated to reducing secondhand smoke tobacco exposure in order to protect the health of nonsmokers. We hypothesized that smoke-free policies or regulations reduced tobacco smoking exposure among the general public in an intervention city relative to a comparison city that lacked such policies or regulations. We further hypothesized that tobacco smoking prevalence would decline significantly as result of the smoke-free intervention in public places. However, in acknowledging that our 15-month intervention period might be too brief to impact this prevalence, we secondarily hypothesized that smoke-free regulations improved health beliefs, attitudes, and behavior among Chinese urban residents concerning the hazards of secondhand tobacco smoke.

## 2. Methods

### 2.1. Setting

The Zhejiang Province People’s Congress ratified smoke-free regulations in public places (SFR) in Hangzhou, our intervention city, on 27 November 2009. With a population of 6.72 million, Hangzhou (HZ) is an economically developed city (*per capita* gross regional product: 61,258 Yuan) and popular tourist destination that is located in Zhejiang Province in southeast China [16]. Its smoking regulations were implemented on 1 March 2010. Under their terms, smoking was completely banned in kindergartens, nursery schools, cinemas, music halls, libraries, exhibition halls, stadiums, public transportation, meeting rooms, elevators, tunnels, and other indoor areas in schools and hospitals. It was partially banned in dance and entertainment facilities, markets, shopping malls, bus and railway station waiting rooms, offices, conference rooms, restaurants in government and nongovernmental organization (NGO) buildings, hairdressers and massage parlors, internet cafes, hotels, and restaurants [17]. Also a major city in Zhejiang Province, our comparison city is Jiaxing (JX). It has no SFR and is located only 90 km from Hangzhou.

### 2.2. Study Design

The study comprised two cross-sectional surveys, which we administered simultaneously in both the intervention and comparison cites. The first wave occurred over the period 10–20 October 2009 and the second over the period 10–20 June 2011, representing a time span of 20 months.

### 2.3. Sampling and Sample

Both surveys utilized a multi-stage sampling design. In Stage 1, we selected our intervention and comparison cities. The intervention city, Hangzhou, was an early adopter of SFR in China. In Stage 2, we randomly selected two residential districts within each city, and then randomly selected four communities within each district. In Stage 3, the Community Committee Office randomly sampled households in each community. These households were distributed across each community in approximate proportion to their estimated overall distribution across each city cluster of communities. Participants were selected independently in each wave, while the districts and the communities were identical in both surveys. The inclusion criterion was being a resident aged 15 years or older. We selected for interview one eligible resident from each household based on birthdate closest to interview date. A total of 80–100 participants were randomly selected within each community, yielding a total of 658–800 participants for each city in each wave of the survey. While based on a power analysis, our sample size was informed by our prior studies and similar studies [18,19,20,21]. Our proposed sample size (minimum 658 participants per city) would enable us to detect an odds ratio (OR) of 1.5 between cities, at 90% power, assuming a reference rate of 70% in a simple logistic model without considering a within-clustering (*i.e.*, district or community) effect. An OR of 1.6 could be detected at 90% power under a scenario where questionnaire completion was 80%. 

### 2.4. Methods of Data Collection

We scheduled a face-to-face individual questionnaire survey once an eligible individual was identified and agreed to study participation. All surveys were conducted by means of a structured, self-administered questionnaire. Surveyors were second-year medical graduate students or fourth-year medical students. Each surveyor received one-day training on the study protocol and survey procedures. Questionnaires were administered privately to participants in their home or in a quiet place, such as a backyard or community park. Appointments were scheduled through a community organization, and were rescheduled as necessary. Upon receiving instructions from assistants, participants were asked to fill out a questionnaire of approximately 30 min’ duration. Each participant was afforded an opportunity to ask or seek clarification of questions regarding the survey or questionnaire items, and given adequate time for completion.

We employed a common survey protocol across the two study cities in order to assure homogeneity of the interview and data collection process. The protocol was approved by the Ethics Committee at the Medical Center, Zhejiang University, and we obtained informed written consent from all participants prior to interview. Possessing acceptable psychometric properties, our data collection procedures have been extensively employed in Chinese smoking research [18,19,20,21].

### 2.5. Measures

We utilized a questionnaire to tap selected sociodemographic characteristics (birthdate, gender, ethnicity, level of education, marital status, and occupation) of participants, and also their beliefs, awareness, attitudes, and behavior concerning tobacco smoking and secondhand smoking exposures. The sociodemographic data is summarized in Table 1.

**Table 1:** Sociodemographic characteristics of the study sample.

		N (%)	HZ (Time 1)	HZ (Time 2)	JX (Time 1)	JX (Time 2)	p-value *
			n (%)	n (%)	n (%)	n (%)	
Total		2,867	800	669	740	658	
*Age (years)*							
	<25	226 (7.9)	34 (4.3)	62 (9.3)	64 (8.7)	66 (10.0)	0.328
	25-	666 (23.2)	195 (24.4)	152 (22.7)	151 (20.4)	168 (25.5)	
	35-	620 (21.6)	156 (19.5)	134 (20.0)	160 (21.6)	170 (25.8)	
	45-	489 (17.1)	170 (21.2)	128 (19.1)	90 (12.2)	101 (15.4)	
	55-	866 (30.2)	245 (30.6)	193 (28.9)	275 (37.2)	153 (23.3)	
*Gender*							
	Male	1,648 (57.5)	472 (59.0)	387 (57.8)	411 (55.5)	378 (57.5)	0.966
	Female	1,219 (42.5)	328 (41.0)	282 (42.2)	329 (44.5)	280 (42.6)	
*Ethnicity*							
	Han	2,832 (98.8)	786 (98.2)	660 (98.7)	731 (98.8)	655 (99.5)	0.239
	Other	35 (1.2)	14 (1.8)	9 (1.3)	9 (1.2)	3 (0.5)	
*Education*							
	Elementary or lower	315 (11.0)	84 (10.5)	89 (13.3)	81 (11.0)	61 (9.3)	0.100
	Junior high school	908 (31.7)	252 (31.5)	223 (33.3)	278 (37.6)	155 (23.6)	
	High school	702 (24.5)	196 (24.5)	155 (23.2)	193 (26.1)	158 (24.0)	
	College and above	942 (32.9)	268 (33.5)	202 (30.2)	188 (25.4)	284 (43.2)	
*Marital status*							
	Never married	444 (15.5)	109 (13.6)	122 (18.2)	120 (16.2)	93 (14.1)	0.480
	Married	2,294 (80.0)	643 (80.4)	518 (77.4)	583 (78.8)	550 (83.6)	
	Divorced/Widowed	129 (4.5)	48 (6.0)	29 (4.3)	37 (5.0)	15 (2.3)	
*Occupation*							
	Managers and clerks	220 (7.7)	80 (10.0)	53 (7.9)	47 (6.4)	40 (6.1)	0.005
	Professionals	199 (6.9)	66 (8.3)	37 (5.5)	32 (4.3)	64 (9.7)	
	Commerce and service	504 (17.6)	129 (16.1)	133 (19.9)	121 (16.4)	121 (18.4)	
	Technical workers	344 (12.0)	68 (8.5)	71 (10.6)	68 (9.2)	137 (20.8)	
	Students or military	322 (11.2)	102 (12.8)	56 (8.3)	94 (12.7)	70 (10.6)	
	Operations	225 (7.9)	38 (4.8)	74 (11.1)	50 (6.8))	63 (9.6)	
	Retired	604 (21.1)	170 (21.3)	164 (24.5)	199 (26.9)	71 (10.8)	
	Other	449 (15.7)	147 (18.4)	81 (12.1)	129 (17.4)	92 (13.9)	

* *p*-values in the table were obtained from the logistical models, and indicate the significance of the interaction of city x time.

We measured general secondhand smoke exposure as exposure of nonsmokers to tobacco smoke for at least 15 minutes daily. Concerning SHS exposure in public places, participants were asked two questions: (1) “Have you been in hospitals or other healthcare facilities, schools, government buildings, and restaurants in the past six months?” (yes/no), and (2) “Have you seen people smoke in these facilities?” Response options for both questions were never/rarely/sometimes/often/always. We formed dichotomous outcome measures using a separation between “rarely” and “sometimes” responses as our cut-point [5,18,19]. Our designated venues were chosen because they were public places that were subjected to smoking bans under the Hangzhou SFR.

We collected self-report data on smoking status and frequency. We defined a “current smoker” as someone who was an active smoker of cigarettes on the day of interview, a “daily smoker” as someone who smoked every day, and an “occasional smoker” as one who smoked on some days [18,19]. Respective dependent variables were daily smoking and occasional smoking. We coded these two outcome measures dichotomously as 0 = smoking and 1 = no smoking.

Two questions measured behaviors about avoiding smoking near others and in public places, respectively. The first question asked, “Do you avoid smoking near others?” Response options were never/rarely/sometimes/often/always. We formed a dichotomous outcome measure using a separation between “rarely” and “sometimes” responses as our cut-point [5,18,19]. The second question on avoidant smoking behavior asked, “Do you smoke in public places?” Response options were never/rarely/sometimes/often/always. Again, we formed a dichotomous outcome measure using a separation between the “rarely” and “sometimes” responses as our cut-point.

We then measured belief and awareness of harm from secondhand smoke (SHS). In measuring *belief* about harm from secondhand smoke (SHS), participants were asked: “Do you think SHS is harmful to health?” Response options were “not harmful/possibly not harmful/uncertain/possibly harmful/harmful.” For analytic purposes, we used a separation between the “uncertain” and “possibly harmful” responses as a cut-point for forming a dichotomous variable. *Awareness* was assessed by the following question for nonsmokers: “Do you care about someone smoking around you?” Response options were “very much/somewhat/not at all.” This outcome measure was dichotomized using a separation between “somewhat” and “not at all” responses as the cut-point [5,19].

In assessing *attitudes* towards tobacco control, we asked participants both about smoking bans in public places andsmoking near women and children. Response options for each question conformed to a 5-point Likert-type scale, 1 (strongly disapprove), 2 (disapprove), 3 (indifferent), 4 (approve), and 5 (strongly approve). We subsequently recoded the responses into dichotomous outcomes using a separation of the “indifferent” and “approve” responses as our cut-point [5,18,19].

Two *behavioral* aspects of tobacco control were addressed in this study: restricting smoking behavior and advising others to quit smoking. The restrictions pertained to households and to workplaces*.* In addressing the household domain, we asked participants the following questions: (1) “What measures have been taken for restricting smoking in your household?” and (2) “What measures have been taken for restricting smoking in your workplace?” Response options for both questions were complete restriction/partial restriction/no restriction) [5,18,19]. We dichotomized our outcome measures as complete *versus* partial or no restriction.

In addressing the issue of advising others to quit smoking, we distinguished, as the intended recipients, household family members from other relatives and friends. Participants were asked two separate questions, each with an accompanying contingent question: (1) “Do any of your household family members smoke cigarettes?” (yes/no), and if yes, (1a) “In the past six months have you advised them to quit smoking?” (yes/no). (2) “Do any of your friends or other relatives smoke cigarettes?” (yes/no), and if yes, (2a) “In the past six months have you advised them to quit smoking?” (yes/no) Family members were defined as cohabitants [5,18,19].

## 3. Data Analysis

All primary dependent variables were dichotomous (Table 2). Key factors (or predictors or independent variables) of interest were city (HZ *vs.* JX), time (Time 1 and Time 2), and their interaction. The primary statistical analysis involved use of a logistic model to assess the association between a dependent variable and the key factors of interest. Both unadjusted and adjusted methods were considered in analyses. The unadjusted method used only the key factors of interest as independent variables in the analyses, while the adjusted method added all of the possible confounders listed in Table 1 as covariates in the logistic models. We applied a general estimating equation (GEE) method in computation, using community as the clustering unit, in order to account for a within-clustering correlation attributable to the complex survey design. For all other categorical variables listed in Table 1, we used categorical and ordinal logistic models, respectively, in assessing their associations with our key factors of interest. All categorical variables were summarized in terms of frequency (and percent) for each city and time. Comparisons of dependent variables between cities or times were performed by means of odds ratios estimated from the logistic models using the adjusted method (Table 3). We employed SAS 9.3 software to conduct the statistical analyses (SAS, Cary, NC). *P*-values ≤ 0.05 indicated statistically significant differences.

## 4. Results

At Time 1 (pre-intervention), we contacted 842 and 776 households in HZ and JX, respectively, and 813 and 753 agreed to participate. Eight hundred (95%) and 740 (95%) eligible participants from these respective households completed the questionnaire. At Time 2, we contacted 704 and 695 households in HZ and JX, respectively. Of these households, 679 and 663 agreed to participate, and 669 (95%) and 658 (95%) of the eligible residents completed the questionnaire. In general, characteristics of the four samples resembled corresponding population characteristics, and only occupation showed significant variation across these groups (Table 1).

Table 2 shows prevalence data across time and treatment groups for dependent variables pertaining to secondhand tobacco smoke exposure in public places; smoking type and behavior; belief and awareness concerning harm from secondhand smoke exposure; personal attitudes towards public smoking bans and smoking near women and children; tobacco control behavior that encompassed smoking restrictions in households and workplaces; and smoking by family, friends, and relatives; and dependent variables related to secondhand smoke exposure.

**Table 2:** Smoking and tobacco control variables by time of survey.

Outcome Variables		Time 1	Time 2	X^2^ *p* *
		N X %	N X %	
**Secondhand smoke (SHS) exposure**				
*General*				
	Hangzhou	498 173 34.7	467 139 29.8	7.49 0.0578
	Jiaxing	458 145 31.6	432 111 25.7	
*Healthcare facilities*				
	Hangzhou	566 172 30.4	381 97 25.5	25.43 <0.0001
	Jiaxing	575 191 33.3	574 150 26.1	
*Schools*				
	Hangzhou	448 154 34.4	308 85 27.6	9.65 0.0218
	Jiaxing	471 163 34.6	448 123 27.5	
*Government buildings*				
	Hangzhou	475 247 52.0	263 83 31.6	48.10 <0.0001
	Jiaxing	483 273 56.5	419 175 41.8	
*Buses or taxis*				
	Hangzhou	739 232 31.4	583 101 17.3	14.72 <0.0021
	Jiaxing	671 217 32.3	618 158 25.6	
*Restaurants*				
	Hangzhou	704 546 77.6	546 338 61.9	17.74 0.0005
	Jiaxing	662 530 80.1	605 448 74.1	
**Smoking type and behavior**				
*Daily smoking*				
	Hangzhou	800 197 24.6	669 158 23.6	0.55 0.9072
	Jiaxing	740 198 26.6	658 158 24.6	
*Occasional smoking*				
	Hangzhou	800 105 13.1	669 44 6.6	11.44 0.0096
	Jiaxing	740 83 11.2	658 68 10.3	
*Avoiding smoking near others*				
	Hangzhou	800 164 20.5	669 150 22.4	17.25 0.0005
	Jiaxing	740 174 23.5	658 138 20.9	
*Avoiding smoking in public places*				
	Hangzhou	800 673 84.1	669 617 92.2	12.83 0.0050
	Jiaxing	740 622 84.0	658 566 86.1	
**Belief in harm from SHS **				
	Hangzhou	800 726 90.8	669 621 92.8	19.28 0.037
	Jiaxing	740 685 92.6	658 578 87.8	
**Awareness of harm from SHS **				
	Hangzhou	498 445 89.4	467 432 92.5	28.38 <0.0001
	Jiaxing	459 423 92.2	432 402 93.1	
**Attitudes toward tobacco control**				
*Banning smoking in public places*				
	Hangzhou	800 624 78.0	669 547 81.8	74.21 <0.0001
	Jiaxing	740 594 80.3	658 563 85.6	
*Not smoking near women and children*				
	Hangzhou	800 698 87.3	669 600 91.2	73.45 <0.0001
	Jiaxing	740 686 92.7	658 599 91.0	
**Tobacco control behavior**				
*Quitting advice for family members*				
	Hangzhou	514 106 20.6	315 273 86.7	181.70 <0.0001
	Jiaxing	532 160 30.1	422 96 22.8	
*Quitting advice for friends or relatives*				
	Hangzhou	713 621 87.1	599 467 78.0	27.86 <0.0001
	Jiaxing	712 569 79.9	640 521 81.4	
*Household smoking restrictions*				
	Hangzhou	800 694 86.8	669 586 87.6	0.43 0.9348
	Jiaxing	740 645 87.2	658 565 85.8	
*Workplace smoking restrictions*				
	Hangzhou	586 218 37.2	418 213 51.0	36.18 <0.0001
	Jiaxing	585 195 33.3	589 406 68.9	

* *p*-values were obtained from unadjusted logistic models and indicate significance of city × time interactions.

Time and treatment were associated with changes in secondhand smoke exposure in all of our specified kinds of public places, occasional smoking prevalence, avoidance of smoking near others and in public places, belief in harm from secondhand smoking exposure, awareness of such harm, and attitudes towards public smoking bans, dispensation of quitting advice to family members and friends or other relatives, and workplace smoking restrictions.

The multiple regression analyses identified a statistically significant association between smoke-free regulations in Hangzhou and a reduction in secondhand smoke exposure in government buildings, buses or taxis, and restaurants following the intervention (Table 3). Both the intervention and time variables showed positive associations with dispensation of quitting advice to family members, and the presence of complete workplace smoking restrictions, respectively. There also was a significant interaction effect between intervention and time groups for dispensing quitting advice to family members; adjusted odds ratios (OR) were 2.32 (95% CI:1.35, 4.00) for the former and 2.94 (95% CI:1.61, 5.26) for the latter, and 0.52 (95% CI:0.37, 0.77) for the interaction term.

**Table 3:** Multiple regression results for assessing respective associations between the smoke-free regulation intervention and outcome variables, adjusting for exposure to smoke-free regulations and time.

Outcome Variables		Regulation Exposure	Time
		OR 95% CI	OR 95% CI
*Secondhand smoke exposure*			
	General exposure	0.50 0.24 1.03	0.65 0.42 1.09
	Healthcare facilities	1.40 0.98 2.02	0.71 * 0.53 0.96
	Schools	1.33 0.96 1.85	0.68 * 0.48 0.97
	Government buildings	1.72 ** 1.32 2.25	0.43 ** 0.33 0.56
	Buses or taxis	1.26 * 1.05 1.49	0.56 ** 0.47 0.67
	Restaurants	1.23 ** 1.09 2.03	0.45 ** 0.22 0.89
*Smoking type and behavior*			
	Daily smoking	1.14 0.89 1.46	0.90 0.69 1.17
	Occasional smoking	1.15 0.76 1.76	0.61 ** 0.42 0.90
	Avoiding smoking near others	1.13 0.98 1.30	1.52 ** 1.17 1.98
	Avoiding smoking in public places	1.30 0.97 1.74	1.23 * 1.06 1.42
*Belief in harm from SHS*		0.90 0.65 1.29	0.92 0.65 1.29
*Awareness of harm from SHS*		0.97 0.86 1.24	1.42 * 1.05 1.91
*Attitudes toward tobacco control*			
	Banning smoking in public places	1.08 0.55 1.04	1.59 ** 1.17 2.18
	Smoking near women and children	1.29 0.88 1.88	1.61 ** 1.13 2.19
*Tobacco control behavior*			
	Quitting advice for family members	4.52 ** 2.39 8.52	5.88 ** 3.45 14.00
	Quitting advice for friends or relatives	1.27 0.98 1.66	1.73 0.92 1.50
	Household smoking restrictions	0.93 0.67 1.28	0.96 0.68 1.35
	Workplace smoking restrictions	1.25 * 1.03 1.54	2.69 ** 2.10 3.46

* and ** indicate significant odds ratios at *p* < 0.05 and 0.01, respectively.

## 5. Discussion

The primary aim of legislation on smoke-free environments is to protect the population, nonsmokers and smokers alike, from the deleterious effects of exposure to secondhand tobacco smoke. Our study was the first to analyze the effectiveness of SFR on secondhand smoke exposure, smoking behavior, and beliefs and attitudes about tobacco control in Chinese cities. It showed that SFR inhibited secondhand smoke exposure in several venues where smoking bans were in effect, namely, government buildings, buses or taxis, and restaurants. Thus, the Hangzhou SFR has restricted SHS exposure to some degree in public places, a positive impact which endured at least 15 months beyond implementation of the intervention. This finding underscores the need to advocate for implementation of SFR in other cities and regions in China. Results from Table 2, Table 3 are similar, which implies that any confounding effects from demographic characteristics are limited.

Our analysis indicated that the impact of the Hangzhou SFR was limited. Exposure to secondhand tobacco smoke remains common in public places. Adoption of a stricter SFR or tobacco control laws is essential for optimal benefits to accrue. Accordingly, we suggest that the Hangzhou SFR be revised to meet the requirements of the Framework Convention on Tobacco Control (FCTC). Moreover, planned SFR legislation in other cities should consider our documented limitations of the Hangzhou SFR. The Hangzhou SFR lacks both specific enforcement guidelines and strong penalties for violation of smoke-free regulations. Thus, the regulations cannot completely prevent secondhand smoke exposure. Efforts to restrict smoking in public places in China should emphasize a total ban, while simultaneously raising public awareness of the perils of secondhand smoke. Article 8 (Protection from Exposure to Tobacco Smoke) of the FCTC outlines specific measures and approaches for reducing population-wide tobacco smoke exposure. National and local ordinances and regulations to reduce secondhand tobacco smoke exposure have been implemented in many of the more developed countries. Less developed countries are now beginning to follow suit. A recent study found that approximately 82% (95% CI: 81.1–82.5%) of participants supported banning smoking in public places in China [6]. National and local authorities can enact public policies to protect people from exposure to secondhand tobacco smoke, and in so doing protect children from smoking-related morbidity and mortality. There is support for comprehensive laws and implementation and penalties from the success in Ireland which was the first country to introduce comprehensive smoke-free laws [22], it is the best choice to complement national comprehensive smoke-free policy in China for preventing secondhand tobacco smoke exposure.

Numerous studies have reported that smoke-free laws may substantially reduce smoking prevalence [6,7,8]. From the viewpoint of social norms, the behavior of people is influenced by their perceptions of what is “normal” or “typical.” Smoke-free laws may alter social norms and lead people to change their beliefs, awareness, attitudes, and practices concerning smoking [7,9,11]. Behavioral susceptibility theory argues that if a given behavior becomes inconvenient or difficult, this behavior will gradually decline [10,11]. While smoke-free laws increase this possibility for smoking, such an outcome was not manifest in our study. Our finding emulated a Canadian finding which showed that smoke-free legislation exerted no impact upon smoking prevalence, but was associated with statistically significant reductions in exposure to secondhand tobacco smoke in public places [23]. The SFR failed to change tobacco control beliefs, awareness, attitudes, and behavior in Hangzhou residents, except with respect to participants advising family members to quit smoking. A possible explanation for this failure is that the SFR was too limited in its coverage of public places, and that implementation was weak [23]. Furthermore, unlike in industrial societies, agrarian social mores persist in China, popular awareness of legal constraints is low, and compliance with laws is typically weak [4,11,13,14]. Our study showed that only 28% of Hangzhou residents believe that implementation of their city SFR is satisfactory. This would suggest that adoption and enforcement of SFR policies are incomplete in Hangzhou. Insufficient public education, both preceding and during the period in which regulations were operant, is another possible determinant of the failure of the SFR to change tobacco control beliefs, awareness, attitudes, and behavior. Public education can be effective in raising public consciousness and changing unfavorable beliefs and attitudes concerning tobacco control [13,14]. To attain such outcomes, it is imperative that smoke-free regulations and laws be enforced. Ideally, efforts to restrict smoking in public places should culminate in a universal smoking ban, as well as raise public awareness of the perils of secondhand smoke. However, smoking remains a Chinese norm. People smoke during social interactions and work as a matter of course. They offer cigarettes to each other as commodities, and prohibition of smoking is associated with loss of face. Consequently, public education should reflect the twin needs to change the smoking norm and inculcate positive attitudes towards tobacco control [24].

Significant changes emerged in SHS exposure and tobacco control beliefs, awareness, attitudes, and behavior among urban residents across time, both in the intervention and comparison city. This finding plausibly reflects joint tobacco-control efforts of the Chinese government, internal social organizations, and international organizations. Currently, more than 10 cities have smoke-free movements, and nationwide campaigns have been waged to ban smoking on university campuses and in hospitals [5,25]. We believe that, collectively, these tobacco control initiatives possess strong potential for changing personal beliefs, awareness, and attitudes in China about the perils of secondhand smoke, and subsequently for changing smoking behavior.

Time 2 *versus* Time 1 differences were very striking concerning both in dispensing quitting advice to family members and in restricting workplace smoking. Central to Chinese culture is the high value placed upon the importance of family at the individual, as well as the societal level. Indeed, family values stress the importance of a collective quality in the everyday life and behaviour of the individual, and a strong sense of personal obligation and responsibility to family is a cherished virtue [26]. In this context, the family may perceive smoking as a threat to the health of the smoker and themselves, and family members may assume responsibility for its prevention. One of our prior studies found that familial support was the leading determinant of a smoker attempting to quit [26]. Environmental cues, most notably the tobacco control movement and public education, can induce familial opposition towards smoking by family members. However, we found a significant negative interaction effect between the intervention and time groups regarding quitting advice. Implying a need for reinforcement, the positive impact of tobacco regulations upon dispensing such advice attenuates with time.

Our research has five main limitations. First, the findings may not be generalizable because our study included only one SFR city. Secondly, our assessment of the SFR intervention was confined to two time points. It would be useful to monitor adoption and enforcement of these regulations over time, and to conduct a Time 3 survey in order to detect any dramatic improvements. With three time points, we could also conduct a dose-response analysis. Thirdly, we did not collect data on such salient variables in secondhand smoke exposure as air quality and health impact. The final study limitation, we assessed smoking status through self-report. Such assessment may introduce information bias. On the other hand, self-reported data are the conventional instrument for population-based smoking surveys [1,8,20], We consider that the appropriateness of our data is reinforced by the evidences that showed self-report bias in smoking research is minimal [27,28]. Since smoking represents normative behavior for adults in China, social inhibition of accurate reporting is a plausible but minimal concern. It should be mentioned that this paper only included self-report variables for the limited space, however, our study covered both observation and self-report indicators, and the results were consistent on both ones.

## 6. Conclusions

We found that the Hangzhou SFR was associated with an inhibition of secondhand tobacco smoking exposure in certain kinds of public places. However, the impact was limited. Our study suggests that effective tobacco control in China will require comprehensive laws implemented fully and supported by penalties, and a combination of strong public health education.
